# A Random Forest-Based Genome-Wide Scan Reveals Fertility-Related Candidate Genes and Potential Inter-Chromosomal Epistatic Regions Associated With Age at First Calving in Nellore Cattle

**DOI:** 10.3389/fgene.2022.834724

**Published:** 2022-05-18

**Authors:** Anderson Antonio Carvalho Alves, Rebeka Magalhães da Costa, Larissa Fernanda Simielli Fonseca, Roberto Carvalheiro, Ricardo Vieira Ventura, Guilherme Jordão de Magalhães Rosa, Lucia Galvão Albuquerque

**Affiliations:** ^1^ Department of Animal Science, School of Agricultural and Veterinary Sciences, Sao Paulo State University (UNESP), Jaboticabal, Brazil; ^2^ National Council for Scientific and Technological Development (CNPq), Brasília, Brazil; ^3^ Department of Animal Nutrition and Production, School of Veterinary Medicine and Animal Science, University of São Paulo, Pirassununga, Brazil; ^4^ Department of Animal and Dairy Sciences, University of Wisconsin-Madison, Madison, WI, United States

**Keywords:** beef cattle, candidate genes, ensemble learning, fertility traits, non-parametric methods, physiological epistasis

## Abstract

This study aimed to perform a genome-wide association analysis (GWAS) using the Random Forest (RF) approach for scanning candidate genes for age at first calving (AFC) in Nellore cattle. Additionally, potential epistatic effects were investigated using linear mixed models with pairwise interactions between all markers with high importance scores within the tree ensemble non-linear structure. Data from Nellore cattle were used, including records of animals born between 1984 and 2015 and raised in commercial herds located in different regions of Brazil. The estimated breeding values (EBV) were computed and used as the response variable in the genomic analyses. After quality control, the remaining number of animals and SNPs considered were 3,174 and 360,130, respectively. Five independent RF analyses were carried out, considering different initialization seeds. The importance score of each SNP was averaged across the independent RF analyses to rank the markers according to their predictive relevance. A total of 117 SNPs associated with AFC were identified, which spanned 10 autosomes (2, 3, 5, 10, 11, 17, 18, 21, 24, and 25). In total, 23 non-overlapping genomic regions embedded 262 candidate genes for AFC. Enrichment analysis and previous evidence in the literature revealed that many candidate genes annotated close to the lead SNPs have key roles in fertility, including embryo pre-implantation and development, embryonic viability, male germinal cell maturation, and pheromone recognition. Furthermore, some genomic regions previously associated with fertility and growth traits in Nellore cattle were also detected in the present study, reinforcing the effectiveness of RF for pre-screening candidate regions associated with complex traits. Complementary analyses revealed that many SNPs top-ranked in the RF-based GWAS did not present a strong marginal linear effect but are potentially involved in epistatic hotspots between genomic regions in different autosomes, remarkably in the BTAs 3, 5, 11, and 21. The reported results are expected to enhance the understanding of genetic mechanisms involved in the biological regulation of AFC in this cattle breed.

## 1 Introduction

Adaptation to tropical environments and resistance to parasites are attributes that make Nellore cattle (*Bos indicus*) an important genetic resource for Brazilian pasture-based beef production systems. Nonetheless, *B. indicus* breeds generally present lower reproductive efficiency compared to taurine cattle (Abeygunawardena and Dematawewa, 2004; Sartori et al., 2010), which limits the selection pressure on replacement heifers. It is known that the efficiency of reproductive performance is intimately associated with beef cattle industries’ profitability since a large proportion of the production system costs is due to the cow’s maintenance in the herd (Malhado et al., 2013). Hence, attaining high fertility rates is a key component for reducing costs in beef production systems.

Age at first calving (AFC) is one of the most common selection criteria for fertility in beef cattle breeding programs, among other reasons, because it can be easily measured and contributes to improving heifers’ sexual precocity. Identifying genes associated with the maintenance of reproductive functions is therefore of paramount importance for enhancing the understanding of the AFC genetic basis, which may have practical implications in designing more efficient breeding strategies to improve fertility rates in Nellore cattle populations. Technological advances and cost reduction of high-throughput genotyping technologies have popularized genome-wide association studies (GWAS), which have contributed to revealing several candidate genes for fertility-related traits in beef cattle over recent years (Melo et al., 2016; Teixeira et al., 2017; Nascimento et al., 2018).

Generally, methodologies employed for scanning genomic regions associated with complex traits in livestock capture individual *loci* effects assuming either infinitesimal contribution or locus-specific variance assigned by different *a priori* distributions (Schmid and Bennewitz, 2017). Despite the conceptual differences, most state-of-art GWAS methods assume only additive gene action for the marker effects, since the additive variance is the genetic component that accounts for the heritable resemblance between relatives for quantitative traits. Paradoxically, under some circumstances, the additive genetic variance is not solely attributable to additive signals and can be viewed as an emergent property of non-additive gene action (Cheverud and Routman, 1995; Hill et al., 2008; Mackay 2013; Huang and Mackay, 2016; Sackton and Hart, 2016). Specifically, even if the variance of the target response variable is expected to be additive, one can expect that epistatic interactions at the level of gene action play an essential role in biological pathways and gene networks, necessary for gene regulation and expression (Phillips, 2008). This implies that additional biological information regarding the trait genetic architecture can be learned from genome-wide scans considering inter-locus interaction effects.

Some studies have been focused on applying machine learning methods (ML) to identify potential causal variants using genome-wide data, especially for human diseases (Szymczak et al., 2009; Goldstein et al., 2010). ML requires minimal or no assumptions about the biological mechanisms governing complex traits, which allows capturing hidden patterns from high-dimensional data (Libbrecht and Noble, 2015). Thus, ML may offer a general framework for unrevealing potential novel causal variants when the true genetic nature underlying the associations between phenotype and markers is unknown and complex. For this purpose, the Random Forest (RF) is one of the most popular learning algorithms. The RF permutation-based variable importance measures provide an intuitive and straightforward approach for selecting and ranking relevant predictors (e.g., single nucleotide polymorphisms—SNPs), while adaptatively dealing with interaction among explanatory variables (Chen and Ishwaran, 2012; Yao et al., 2013). These appealing features may contribute to enhancing our knowledge about the biological mechanisms underlying the expression of complex traits. Nevertheless, applications of the RF to identify genomic regions for reproductive traits in beef cattle are still scarce. This study aimed to perform a GWAS using the RF approach for scanning candidate genes for AFC in Nellore cattle. Also, potential epistatic effects between the top-ranked markers in the RF analysis were investigated via linear mixed models to unveil the nature of the effects detected within the tree ensemble non-linear structure.

## 2 Materials and Methods

### 2.1 Animals and Phenotypic Data

The phenotypic and pedigree data were obtained from the Alliance Nellore database, which integrates information from Nellore cattle raised in different commercial herds, located in the Southeast, Midwest, and Northeast regions of Brazil. Animals included in the database were born between 1984 and 2015. The reproductive management adopted in those herds involves an in-advance breeding season occurring between February and April, with approximately 60 days in length, in which heifers between 14 and 18 months of age are exposed to reproduction for identifying sexually precocious animals. Heifers that did not conceive in the anticipated breeding season participate along with the other dams in the regular breeding season occurring between November and January.

In this study, the Age at First Calving (AFC) was adopted as a fertility-related trait, obtained as the difference in days between the date of first calving and the dam birth date. The contemporary groups (CG) comprised animals born in the same herd, year, and season, and which were raised in the same management group at weaning and yearling. A data editing step was performed, in which animals with records deviating ±3.5 standard deviations from the CG mean were excluded from the dataset. Further, CG with less than five observations were not considered. A mixed model approach was used to compute the estimated breeding values (EBV) for AFC, considering the following model:

y=Xβ+Za+e

in which **y** is a vector of observed phenotypes; β is the vector of fixed effects for CG; **a** is the vector of random additive genetic effects; **X** and **Z** are incidence matrices connecting β and **a** to the observed values, and **e** is the vector of random residuals. It is assumed that **a** ∼ *N* (0, $A\sigma_{a}^{2}$ ) and **e ∼** N (0, $I\sigma_{e}^{2}$ ), where $\sigma_{a}^{2}$ and $\sigma_{e}^{2}$ are the variance components for the additive and residual random effects, respectively; **A** is the numerator relationship matrix and **I** is a diagonal matrix with proper dimension. The number of animals included in the additive relationship matrix was 329,297. The variance components were estimated by Restricted Maximum Likelihood (REML) using the BLUPF90 family programs (Misztal et al., 2018). The narrow-sense heritability (h^2^) for the studied trait was computed as $\;h^{2}=\;\sigma_{a}^{2}/\left(\sigma_{a}^{2}+\sigma_{e}^{2}\right)$ and the EBVs were then considered as response variables in posterior genomic analyses.

### 2.2 Genotype File and Quality Control

The genotyped population was composed of 8,666 Nellore cattle (1,128 bulls, 2,737 cows, and 4,801 calves), which were initially genotyped with either the Illumina BovineHD panel (HD; 4625 animals) or with the GeneSeek Genomic Profiler Indicus HD (GGP75Ki; 4,041 animals), with approximately 777,000 and 75,000 SNPs, respectively, distributed throughout the genome. The lower density panel (GCP75Ki) genotypes were imputed to HD using the FImpute v2.2 software (Sargolzaei et al., 2014), considering all genotyped animals and pedigree information, with an expected accuracy higher than 0.97 (Carvalheiro et al., 2014). After the imputation procedure, only genotyped samples with EBV accuracy higher than 0.30 for AFC (868 bulls and 2,306 cows) were kept. Because of the low EBV accuracy for AFC, the progeny data were not considered in the genome-wide association study. As a quality control (QC) procedure for the genotypic data, non-autosomal, unmapped, or duplicated SNPs were discarded as well as those with call rate <0.98, minor allelic frequency (MAF) < 0.05, and *p*-value lower than 10^−5^ for the Hardy-Weinberg equilibrium test. Only samples with a call rate higher than 0.90 were maintained in the genotypic data. The genotypes file filtering was performed using the R software (R Development Core Team, 2011). After QC, the number of animals and SNPs retained for analyses was 3,174 and 360,130, respectively.

### 2.3 Genome-Wide Association Analysis With Random Forest

#### 2.3.1 Random Forest Algorithm Description

The random forest (RF) is a machine learning method that aggregates complementary information from an ensemble of classification or regression trees trained on different bootstrap samples (animals) drawn with replacement from the original data set (Breiman, 2001). Briefly, let $y_{\left(nx1\right)}$ be a vector of observations for a given trait and $X_{\left(nxp\right)}$ the markers matrix, with *n* representing the number of available samples and *p* the number of SNPs, coded as 0, 1 and 2 for genotypes AA, AB, and BB, respectively. Initially, a bootstrap sample is drawn from this data set and used for training an individual classification or regression tree. At each node of this given tree, a subset of $M_{try}$ variables are drawn randomly from the overall *p* SNPs and evaluated using a recursive binary splitting rule, for which the best predictor variable $X_{j}$ (with j = 1, 2, … , $M_{try}$ ) and the threshold value $t_{k}$ are those which minimize a given loss function. For continuous responses, the squared loss function is commonly adopted. The tree node is partitioned according to the coordinates $\left\{y|X_{j}\leq \;t_{k}\right\}$ and $\left\{y|X_{j}>\;t_{k}\right\}$ originating two child nodes, which are also partitioned using the same splitting rule (evaluating different $M_{try}$ markers at each node). This process is repeated until the tree reaches terminal nodes with homogenous or near homogenous responses (Chen and Ishwaran, 2012). The predicted outcomes of the tree are the most frequent class (for categorical responses) or the average observation (for continuous responses) at terminal nodes. Finally, several trees are built using $N_{tree}\;$ different bootstrap samples of the same size as the original training data, following the same steps, described previously. The tree ensemble information is aggregated for computing final predictions as follows:

$$\;\hat{y}=\;\frac{1}{Ntree}\sum_{b=1}^{Ntree}T\left(X,\psi_{b}\right),$$

where $\psi_{b}$ represents an individual tree architecture in terms of the bootstrap sample, SNPs selected at each node, and terminal node responses.

A particularity of the RF is the out-of-bag data (OOB), which corresponds to the animals not included (roughly 1/3) in the bootstrap sampling for building a specific tree. Since the bootstrap sampling is performed with replacement, the trees are built using random samples of the same size as the original training data. Notice that some observations may appear more than once in the bootstrap sample, whereas others will not be sampled at all (composing the OBB sample for that specific tree). The OOB can be used as an internal validation set for each tree, which allows the computation of the generalization error term (James et al., 2013). In the continuous case, the mean squared error is generally used as the loss function:

$$MSE_{OOB}=\;\frac{1}{N_{OOB}}\sum_{i=1}^{N_{OOB}}{\left(y_{i}-{\hat{y}}_{i}\right)}^{2},$$

in which $N_{OOB}$ is the number of observations in the OOB samples, ${\hat{y}}_{i}$ is the average of predictions for the *i*th animal computed from trees in which this animal was OOB, and $y_{i}$ is the realized value. The $MSE_{OOB}$ is considered an internal validation of the prediction error and can be used for tuning the RF parameters.

Another appealing attribute of this internal validation process is that it can provide variable importance measures (VIM) for each predictor variable composing the regression trees. The most frequently used measure is the permutation-based VIM, which can be internally computed for the *j*th SNP as the average difference between the $MSE_{OOB}$ when the SNP of interest was randomly permuted in the OOB data and the $MSE_{OOB}$ obtained without permutation, considering all trees. SNPs with higher VIM are suggestive of having an association with the phenotype of interest, since permuting a relevant SNP is expected to increase the OOB prediction error (Mokry et al., 2013; Yao et al., 2013). For an SNP that has no association with the response variable, the permutation-based score is expected to be approximately zero. Similarly, negative importance scores indicate that the permutation of the SNP in the OOB data provided lower generalization error; therefore, this SNP does not have importance for prediction.

#### 2.3.2 Random Forest Implementation

The GWAS was performed using the *randomForest* package (Liaw and Wiener, 2002) available for the R software (R Development Core Team, 2011). Because of the $MSE_{OOB}$ stabilized rapidly in previous analyses, the parameter $N_{tree}$ (i.e., the number of trees to grow) was fixed to 1,000. The assessed values for the $M_{try}$ parameter (i.e., the number of SNPs to test at each node) were 1, $\sqrt{p}$ , 0.01p and 0.1p , in which *p* represents the total number of SNPs. The *nodesize* parameter (i.e., the maximum number of observations at the terminal nodes) was set to default (*nodesize* = 5) in all analyses. The parametrization that produced the lowest final $MSE_{OOB}$ was maintained for further analysis. After defining the best RF parameters configuration, five independent analyses were carried out with different initialization seeds. In addition, a standardized importance factor for each SNP was computed by dividing its original permutation-based score $\left(\%IncMSE_{SNPj}\right)$ by the absolute value of the most negative importance score (Szymczak et al., 2016):

$$f_{SNPj}\;=\;\frac{\%IncMSE_{SNPj}}{\left|\min\%IncMSE_{SNP}\right|}$$

To improve the stability of the GWAS results, the importance scores of each SNP were averaged over the five independent RF analyses to compute the final importance scores. A common practice in genome-wide association studies performed with RF is to set the absolute value of the most negative importance score as the threshold for identifying a subset of relevant SNPs (Yao et al., 2013), this would be equivalent to setting $f_{SNPj}=1$ in our study. Nonetheless, to better control the false-positive discovering rate, we set the threshold $f_{SNPj}\geq 3$ to identify the SNPs with the strongest signals, as suggested by Szymczak et al. (2016). The pairwise linkage disequilibrium (LD) for the top-ranked SNPs in the RF algorithm was computed with the *r* ^*2*^ metric using the Gaston R package (Perdry and Dandine-Roulland, 2018). Notice that the *r* ^*2*^ is also used interchangeably as a measure of gametic-phase disequilibrium (GPD) throughout the manuscript for conceptually differentiating associations between unlinked *loci*.

### 2.4 Identification of Candidate Genes and Enrichment Analysis

The identification of candidate genes flagged by SNPs previously selected in the RF analysis was performed using the genome data viewer (https://www.ncbi.nlm.nih.gov/genome/gdv/?org=bos-taurus) from the National Center for Biotechnology Information (NCBI), considering the ARS-UCD1.2 (https://www.ncbi.nlm.nih.gov/assembly/GCA_002263795.2) as the reference map. For gene annotation, it was considered a 500 Kb window (SNP location ±250 Kb) harboring each SNP with $f_{SNPj}\geq 3$ . For overlapping windows, only the SNP with the highest importance factor $\left(f_{SNPj}\right)$ was considered as the reference location. We used the Toppgene software (Chen et al., 2009) for prioritizing the annotated candidate genes according to their functional similarity with a list of genes embedding quantitative trait loci (QTLs) identified for AFC and other fertility traits. The list of cataloged QTLs and genes was retrieved from the Cattle QTLdb repository (Hu et al., 2013). The prioritization analysis considered the information extracted from databases related to gene ontology (biological processes), pathway enrichments, mouse and human phenotypes, and coexpression networks. Furthermore, seeking to provide more insights regarding the biological processes in which the candidate genes are involved, a functional analysis of the annotated gene list was performed using the ClueGo program (Shannon et al., 2003), coupled with the Cytoscape plug-in (Bindea and Mlecnik, 2012).

### 2.5 Inferring the Gene Action of Markers Identified With the Random Forest

The generated hierarchical tree-based structure in the RF algorithm is informative for capturing both additive and non-additive effects, especially SNP-SNP interactions. In this regard, a marker can receive a high importance score if it presents a strong marginal additive effect or if it interacts with other markers to create important predictive patterns. However, the RF importance scores per se do not provide any information on the nature of the genetic effects captured by SNPs selected in the genome-wide scan. To investigate the gene action of markers previously identified with the RF algorithm, the following linear mixed models were fitted:

$$y^{*}=\;1_{n}µ+x_{i}\alpha_{i}+\;Zu+e$$ (M1)

$$y^{*}=\;1_{n}µ+x_{i}\alpha_{i}+x_{j}\alpha_{j}+\left(x_{i}.x_{j}\right)\delta_{ij}+\;Zu+e$$ (M2)

in which $y^{*}$ is the vector of *n* observed response values, $1_{n}$ is a vector of 1’s, $x_{i}$ and $x_{j}$ (with *i* ≠ *j*) are vectors of pre-selected SNPs in the RF analysis $\left(f_{SNPj}\geq 3\right)$ , with genotypes AA, AB and BB coded as 0, 1, and 2, respectively, $\alpha_{i}$ is the additive effect of the *i*th SNP, $x_{i}.x_{j}$ is the vector of pair-wise interactions, $\delta_{ij}$ is the interaction effect between the SNPs *i* and *j*, Z is the design matrix relating individuals to the random animal effect, u is the vector of genomic breeding values (GEBVs) and e is the vector of random residual effects. In the models M1 and M2 it is assumed that the animal and residual random effects follow a normal distribution, with $u∼N\left(0,\;G\sigma_{u}^{2}\right)$ and $e∼N\left(0,\;I\sigma_{e}^{2}\right)$ , where G is a marker-based genomic relationship matrix (VanRaden, 2008) and $\sigma_{u}^{2}$ is the marker additive genetic variance, I is an identity matrix, and $\sigma_{e}^{2}$ is the residual variance.

The models M1 and M2 were fitted multiple times considering each lead SNP individually (M1) and each pairwise combination between two lead SNPs (M2). The variance components were estimated iteratively using the EMMREML R package (Akdemir and Godfrey, 2015). The significance of individual additive effects (M1) and interactions between SNPs pairs (M2) was assessed with a Wald test, considering $\alpha_{i}=0$ and $\delta_{ij}=0$ as null hypotheses. The *p-values* were corrected for multiple testing using false-discovery rate (FDR; Qu et al., 2010) thresholds of 0.1, 0.05 and 0.01. We further investigated the co-expression relationship among the candidate genes surrounded by SNP markers with significant interaction effects through a functional protein-protein interaction (PPI) analysis using the STRING database (Szklarczyk et al., 2014); the PPI network nodes were clustered with the k-means algorithm according with their functional similarity, considering *k* = 5.

## 3 Results

### 3.1 Response Variable Summary Statistics

**FIGURE 1:**
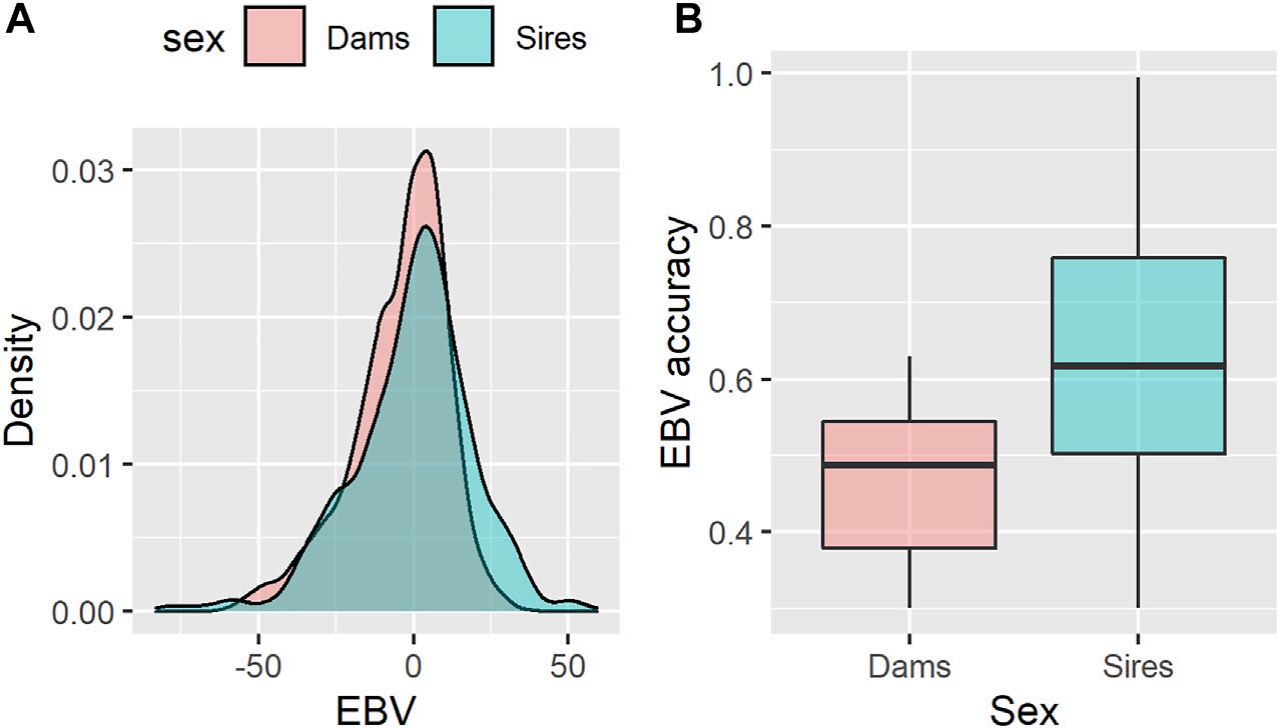
Density plot of estimated breeding values (EBV) for age at first calving in Nellore cattle **(A)** and their respective accuracy **(B)**, according to the sex category.

In the present study, the estimated breeding values (EBVs) for age at first calving (AFC) of 868 sires and 2,306 dams with available genotypes were used as response variables in the RF-based genome-wide association study for AFC. The estimated heritability for AFC was low (0.08 ± 0.005), indicating that this trait is highly influenced by environmental factors and other effects not accounted for in the mixed model analysis. Because of the low heritability value found, a 0.3 cut-off value was imposed for the EBV accuracy to reduce the noise inclusion in the RF analyses. The EBV for both sires and dams showed an approximately normal distribution, lying in similar intervals and with average values of −0.37 ± 19.5, and −4.4 ± 15.4 days, respectively (Figure 1A). On the other hand, the average EBV accuracies were higher for sires (0.63 ± 0.18) than for dams (0.46 ± 0.09), as depicted in Figure 1B. The adoption of EBVs instead of deregressed proofs (dEBV) as response variables was due to the relatively low average reliability of the EBVs (0.28 ± 0.17). In this case, the parental contribution removal would incorporate too much noise during the deregression process. In this scenario, some authors advocate that EBVs would be a reasonable choice for genome-enabled analysis (Morota et al., 2014; Fernandes Junior et al., 2016). Furthermore, preliminary analyses pointed out that the RF algorithm fitted the data better (considering the percentage of variance explained in the OOB data) and consequently had higher SNP ranking power when using the EBVs as response variables rather than dEBVs or phenotypes adjusted for fixed effects (data not shown).

### 3.2 Random Forest Hyperparameters Tuning

**FIGURE 2:**
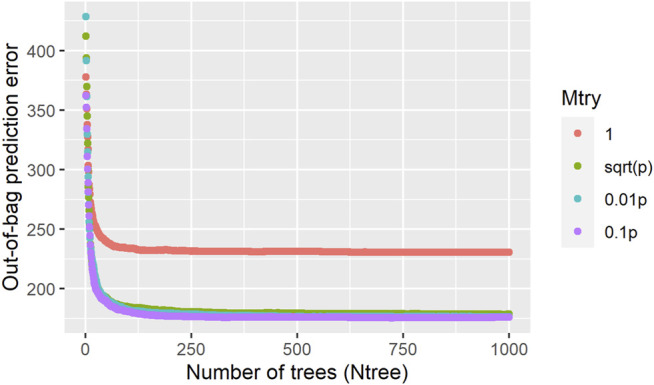
Influence of the random forest hyperparameters (Mtry and Ntree) in the out-of-bag prediction error for age at first calving in Nellore cattle.

The influence of RF parameters on the model predictive performance is presented in Figure 2, it can be seen that the out-of-bag prediction error stabilizes around 200 trees, and 1,000 trees were used as a reliable size for the $N_{tree}$ hyperparameter. Among the assessed values for $M_{try}$ (number of SNP randomly analyzed per tree node), the random single-marker drawing per tree node $\left(M_{try}=1\right)$ produced the worst predictive performance, whereas values $\sqrt{p}$ , 0.01p and 0.1p gave similar results, with $M_{try}=0.1p$ providing a slightly lower OOB prediction error (Figure 2). This parameter controls the trade-off between bias and variance, impacting directly the sparsity of variable importance measures (Goldstein et al., 2010). Since there were no major differences in the OOB error regarding the $M_{try}$ choice, we decided to use a 0.01 value for the subsequent analyses to allow markers with relatively small effects to be selected within the ensemble of trees and for reducing the computational burden of running multiple analyses. Therefore, the genome-wide analyses were performed using $M_{try}=0.01p$ and $N_{tree}=1,000$ for all five RF replicates.

### 3.3 Random Forest-Based Genome-Wide Association Study

**FIGURE 3:**
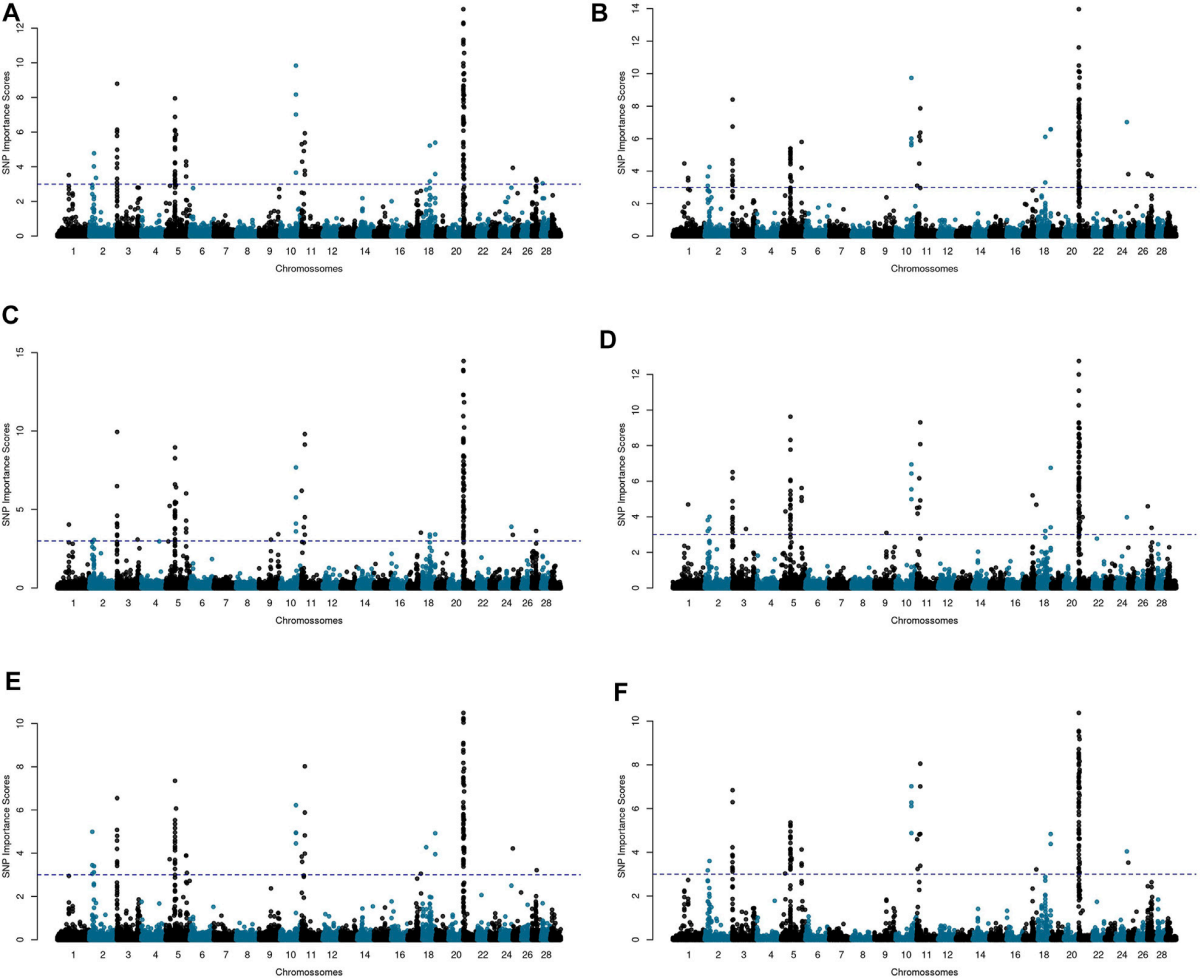
Manhattan plots for age at first calving (AFC) in Nellore cattle considering the relative importance scores computed for each SNP in five independent Random Forest (RF) analyses **(A–E)** and averaged across the RF replicates **(F)**. Negative importance scores were plotted as zero. The blue dashed line corresponds to the threshold value for SNP selection.

To assess the randomness influence on the results, the importance factors $\left(f_{SNPj}\right)$ for all 360,130 SNPs across the 29 *Bos taurus* autosomes (BTA) were obtained in five independent RF-based analyses, initialized with different seeds (Figures 3A–E). In general, the RF replicates highlighted the same genomic regions, providing evidence for the stability of the obtained results. Therefore, we used the average $f_{SNPj}$ across the RF replicates as a reliable summary measure for ranking the genomic markers according to their relative importance for the trait of interest (Figure 3F). Considering a threshold of three for the average $f_{SNPj}$ , the RF approach identified 117 SNPs associated with AFC (Figure 3F), these SNPs were located over 10 BTAs: 2 (2 markers), 3 (11 markers), 5 (29 markers), 10 (4 markers), 11 (7 markers), 17 (1 marker), 18 (2 markers), 21 (59 markers), 24 (1 marker), and 25 (1 marker). The average $f_{SNPj}$ for the identified SNPs was 5.31 and the markers with the highest importance factors were in the BTA 21 (Figures 3A–F). Notice that the higher the $f_{SNPj}$ score the stronger is the SNP predictive importance compared to other markers with spurious signals.

Based on the genomic annotation we found that from the 117 pre-selected SNPs, 6.84% (8) were in exon regions, 33.33% (39) were in intronic regions, and 59.82% (70) were located downstream or upstream of candidate genes. Considering the 250 Kb size (downstream-upstream) window, the selected SNPs with the highest $f_{SNPj}$ harbored 23 non-overlapping genomic regions. Further details such as the marker ID, chromosome and position (Mb), and the importance scores of the 23 lead SNPs flagging the non-overlapped genomic regions are shown in Supplementary Table S1.

**FIGURE 4:**
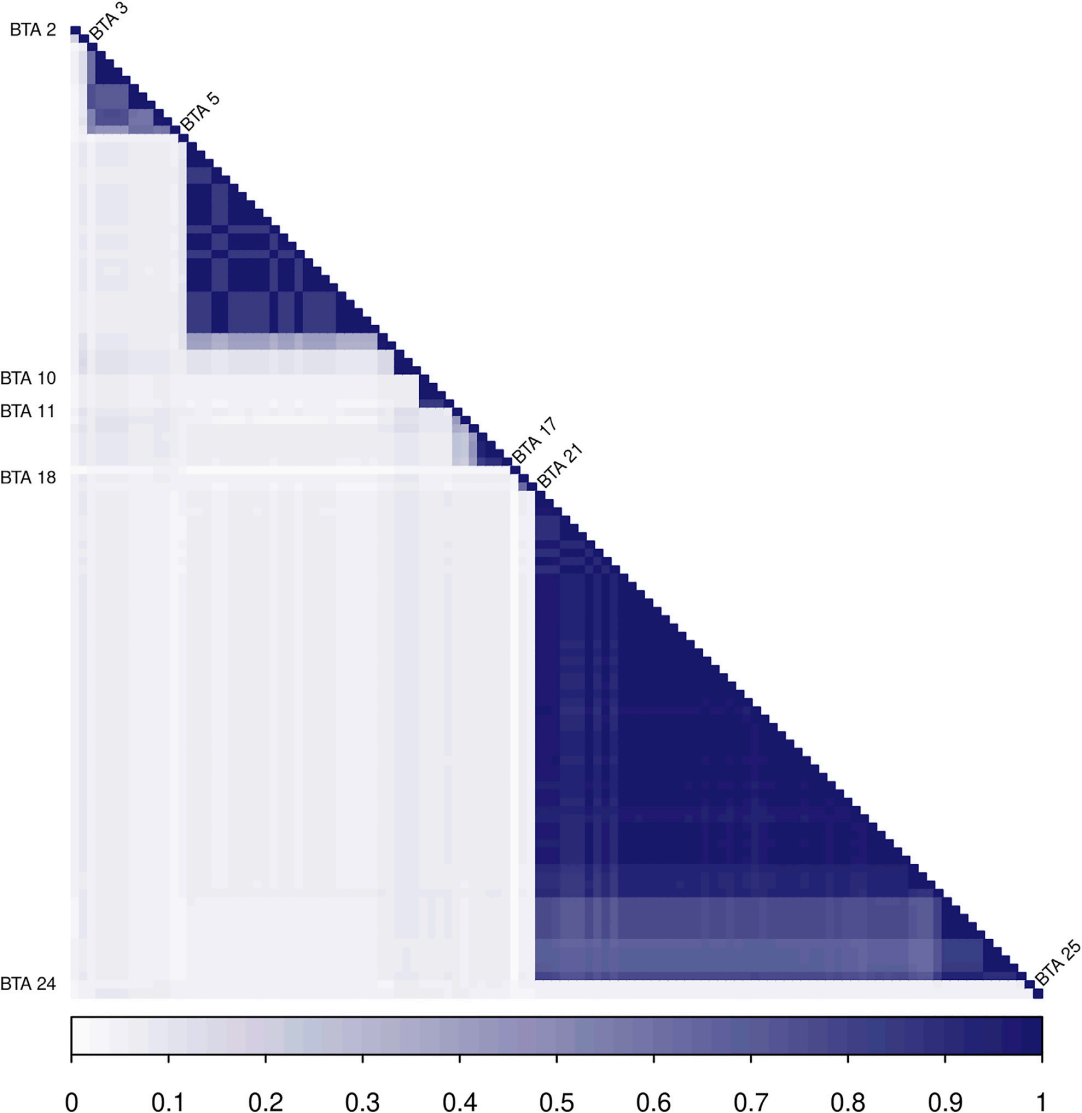
Heatmap of the linkage disequilibrium (LD), measured with the *r* ^2^ metric, among the 117 SNPs identified in the Random Forest analysis. SNPs located in the same chromosome are identified with the BTA label on the left or right sides.

Most of the selected variants in the same BTA are in LD blocks with other relevant neighboring markers (Figure 4), providing evidence for the presence of single or multiple causal mutations in these locations. The highest LD blocks were observed in BTA 3 (total length of 0.95 MB, from 0.262 to 1.207 MB), BTA 5 (total length of 0.23 MB, from 46.02 to 46.25 MB), and BTA 21 (total length of 1.36 MB, from 0.812 to 2.174 MB). The average *r* ^*2*^ in those blocks were 0.71 (0.45–0.99), 0.92 (0.84–0.99), and 0.96 (0.84–0.99), respectively.

### 3.4 Candidate Genes

The full list of candidate genes located within the 250 Kb downstream-upstream interval flagged by the lead SNPs is shown in Supplementary Table S1; 262 genes were annotated and we provided in Supplementary Table S2 the top 30 genes presenting the highest functional similarity with a list of genes previously identified for AFC and other fertility traits. The training list used in the prioritization analysis and the biological processes significantly enriched for these genes is detailed in Supplementary Figure S1. These reference genes are known to be involved in different fertility-related biological processes, such as “developmental growth” (GO0048589), “reproductive process” (GO0022414), “female gonad development” (0008585), “female pregnancy” (GO0007565), “ovulation cycle process” (GO0022602), and “reproductive system development” (GO0061458), which reinforces their appropriateness for being used as training list in the prioritization analysis. One must highlight that some candidate genes identified in the RF analysis pertain to the same family domain of genes included in the training list, e.g., *NLRP5*, *NLRP8*, *NLRP13* (candidate genes), and *NLRP9* (training list), *FUT8* (candidate gene) and *FUT1* (training list), and *SEMA4C* (candidate gene) and *SEMA4A* (training list).

According to the evidence found in the reported literature and the functional analysis results, almost all genomic regions highlighted in this study encompass candidate genes with key roles in male or female fertility, or with growth-associated functions. Considering the prioritization analysis (Toppgene), the functional enrichment (Cytoscape), and the *a priori* evidence reported in the literature, the most promising candidate genes found for AFC are *SP3* (BTA 2), *TBX19*, *CD247*, *CREG1*, *DCAF6*, *ADCY10*, *MPZL1*, *MPC2*, *POU2F1*, *GPR161* (BTA 3), *ATP2B1*, *DYRK2*, *APOBEC1*, *USP15*, *DPPA3*, *NANOG* (BTA 5), *FUT8*, *LMAN2L* (BTA 10), *AFF3*, *ATP6V1B1*, *SEMA4C*, *VAX2*, *TEX261*, *ZAP70* (BTA 11), *MYO18B* (BTA17), *CNOT3*, *NLRP5*, *NLRP8*, *NLRP13*, *LOC107131469*, *LOC107131476*, *LOC107131477*, *LOC107131465*, *PRPF31*, *RPS9*, *TFPT* (BTA18), *MKRN3*, *NDN*, *MAGEL2*, *SNRPN*, *SNURF*, *GABRG3*, *UBE3A* (BTA21), *RAB40C*, *STUB1*, and *AXIN1* (BTA 25).

**FIGURE 5:**
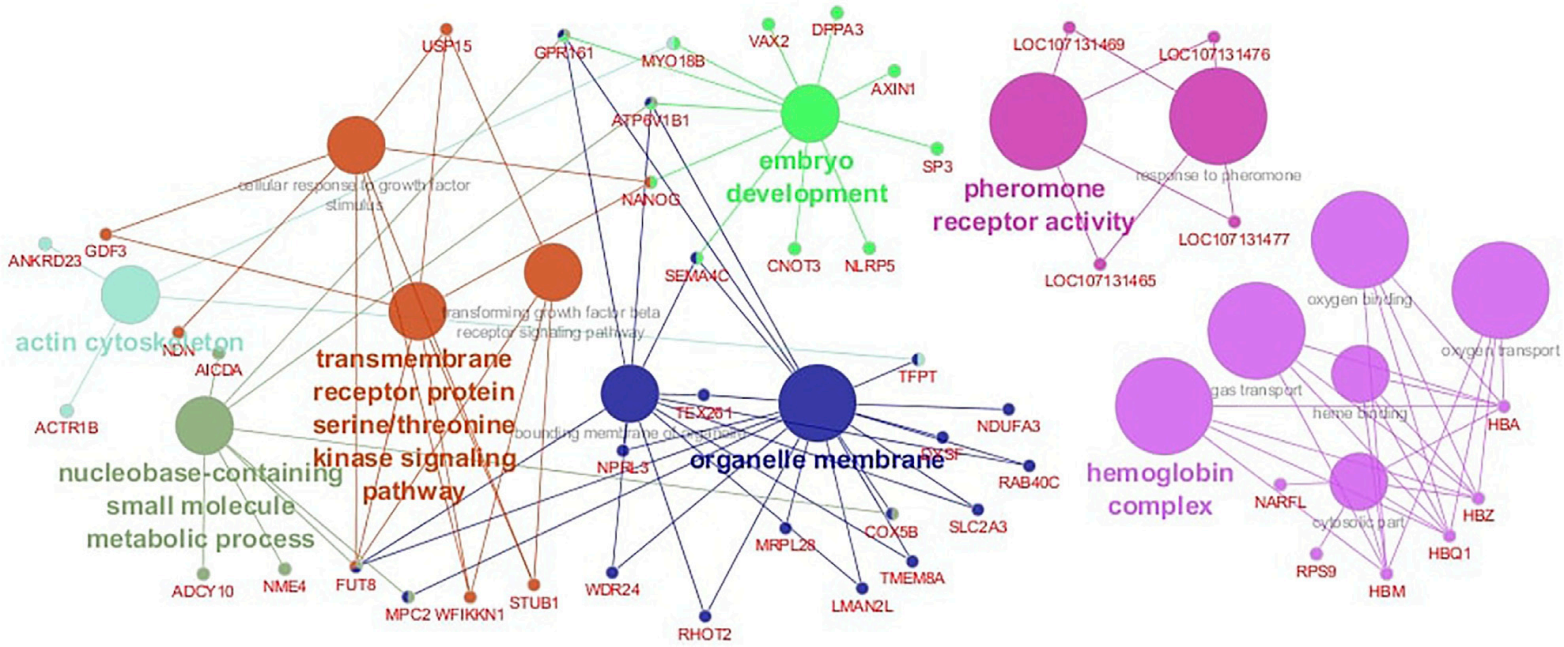
Gene network for Age at First Calving (AFC) in Nellore cattle. Different node colors represent the functional groups in which the candidate genes are involved.

The functional analysis revealed 16 significant biological pathways with which the candidate genes are associated (Figure 5). Some pathways are directly involved in fertility-related processes such as embryo development, pheromone receptor activity, and response to pheromone (Figure 5). Further, several important genes are involved in multiple biological pathways; for instance, the *NANOG* is associated with embryo development and cellular response to growth factor stimulus, and the *GPR161* participates in three different functional groups: “embryo development,” “bounding membrane of organelle,” and “nucleobase-containing small molecule metabolic process” (Figure 5). The functional analysis also evidenced a cluster of genes located in BTA 25 that are involved in biological processes related to hemoglobin functions such as oxygen transporter activity and oxygen binding (Figure 5).

### 3.5 Gene Action Associated With Markers Identified in the Random Forest Analysis

**FIGURE 6:**
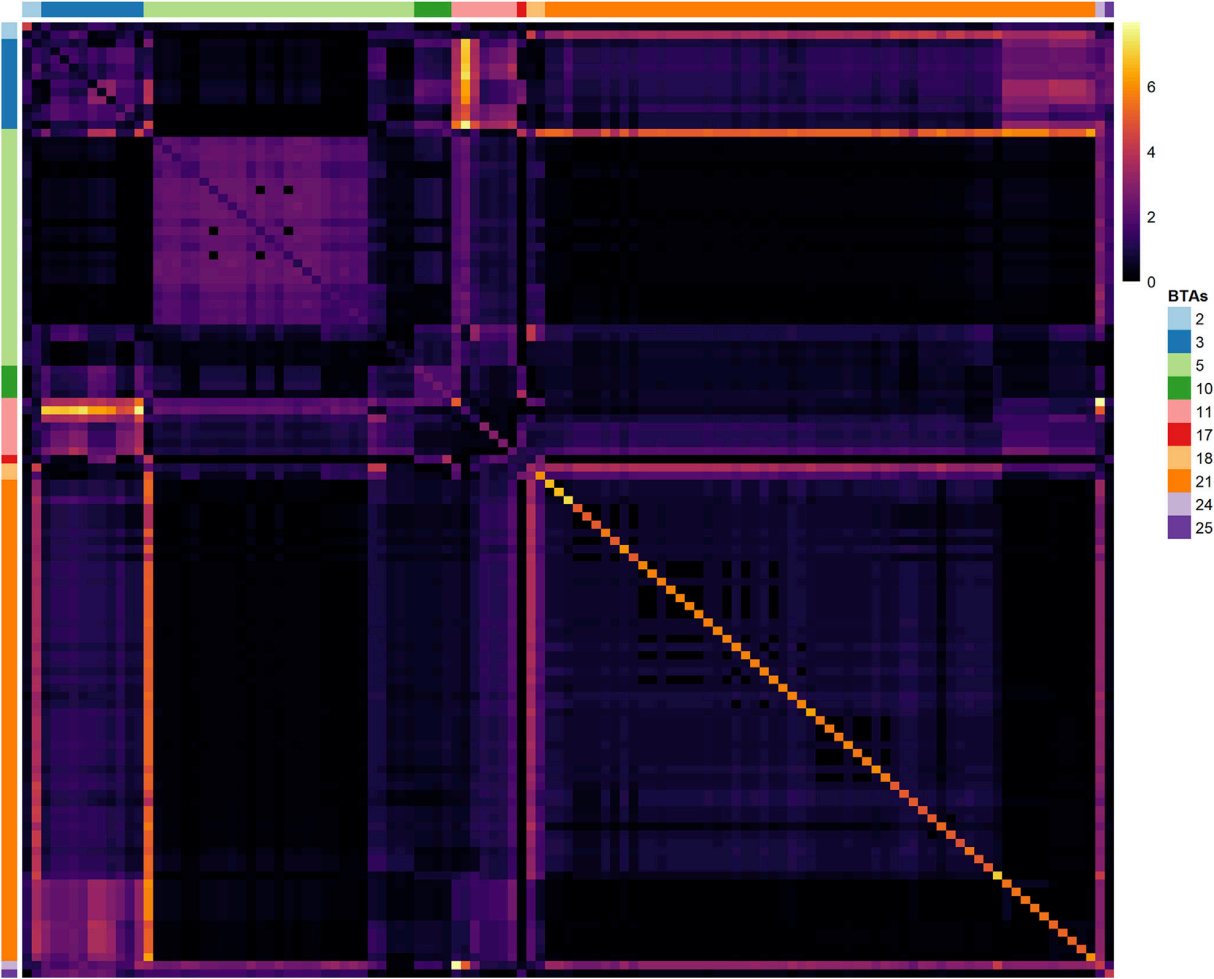
Heatmap of the −log10 (*p*-values) for the marginal (diagonal) and pairwise interaction effects (off-diagonal) computed *via* mixed model analyses for the 117 lead SNPs identified in the Random Forest genome-wide scan for Age at First Calving in Nellore Cattle. The heatmap color key (right side) indicates the significance magnitude for the main and interaction effects in the −log10 (*p*-value) scale. Side color bars (top and left) indicate the Bos taurus autosome (BTA) where each marker is located.

As detailed in the *Material and Methods* section, the gene action associated with the 117 variants identified in the RF analysis was further investigated *via* linear mixed models with pairwise interactions. The correlation coefficient between the $f_{SNPj}$ metric and the −log_10_ (*p*-value) obtained from the single-marker linear regression (M1) was 0.61, indicating only partial agreement between the two approaches. The M1 analyses revealed that several SNPs identified with the RF algorithm do not present strong marginal linear effects, especially those located in BTAs 3, 5, and 11. However, the M2 analyses indicated that several of these markers with weak marginal effects are potentially involved in hotspots of local or inter-chromosomal additive-additive epistatic interactions (Figure 6).

There were 764 epistatic interactions with nominal *p*-values < 0.05, indicating a rejection rate of 11.26% for the null hypothesis $\left(\delta_{ij}=0\right)$ , which is more than twice the expected by chance. The number of significant interactions at FDR thresholds of 0.1, 0.05 and 0.01, were 65, 7, and 0, respectively. These involved 66 SNPs, located in the BTAs 3, 5, 11, 18, 21, and 24. Table 1 presents the BTA number, position (base pairs), and nearest genes for SNPs involved in significant pairwise interactions (FDR threshold of 0.10), considering markers flagging at least one different gene. The gametic-phase disequilibrium (GPD) between interacting markers and *p*-values for the epistatic effects are also provided.

**TABLE 1:** Significant pairwise epistatic effects in the mixed model analyses considering the subset of SNP pre-selected with the Random-Forest-based genome-wide scan for Age at First Calving in Nellore. Only pairs with at least one marker within a different candidate gene are shown.

SNP 1	SNP 2							GPD	*p*-value
BTA	Position (bp)	Nearest Gene	Distance	BTA	Position (bp)	Nearest Gene	Distance		
3	1207469	*CD247*	intron	11	4969068	*AFF3*	intron	0.075	3.0 × 10^−4*^
3	633619	*DCAF6*	intron	11	4969068	*AFF3*	intron	0.101	2.7 × 10^−5**^
3	261913	*TBX19*	exon	11	4969068	*AFF3*	intron	0.075	3.6 × 10^−5**^
3	981696	*MPZL1*	intron	11	4969068	*AFF3*	intron	0.081	1.0 × 10^−4*^
3	1135161	*LOC104971407*	intron	11	4969068	*AFF3*	intron	0.084	7.3 × 10^−4*^
3	1207469	*CD247*	intron	11	2811617	*ANKRD39*	intron	0.068	3.0 × 10^−4*^
5	19823030	*LOC112446651*	108817	18	63426736	*NLRP13*	intron	0.051	7.7 × 10^−4*^
5	19823030	*LOC112446651*	108817	21	5004507	*GABRG3*	intron	0.039	1.0 × 10^−4*^
5	19823030	*LOC112446651*	108817	21	1164298	*MKRN3*	exon	0.043	6.4 × 10^−4*^
5	19823030	*LOC112446651*	108817	21	1195551	*MAGEL2*	exon	0.043	6.4 × 10^−4*^
5	19823030	*LOC112446651*	108817	21	1940618	*SNRPN*	intron	0.043	5.7 × 10^−4*^
11	2811617	*ANKRD39*	intron	24	55609449	*LOC112444186*	133740	0.069	1.0 × 10^−5**^
11	4969068	*AFF3*	intron	24	55609449	*LOC112444186*	133740	0.053	6.2 × 10^−4*^

BTA, *Bos taurus* autosome; Bp, base pairs; GPD, Gametic-phase disequilibrium.

*, **, Significant at the false discovery rate (FDR) threshold of 0.1 and 0.05, respectively.

The most significant interaction effect in the M2 analysis involved a marker in the BTA 11 (2,811,617 bp) within an intronic region of the *ANKRD39* gene, and a marker in the BTA 24, located 133,740 bp downstream of an uncharacterized gene (Table 1). Interestingly, another marker also located in the BTA 11 (4,969,068 bp), at an intronic region of the gene *AFF3*, interacts with markers at intron or exon regions of multiple genes in the BTA 3, namely, *CD247*, *DCAF6*, *TBX19*, and *MPZL1*. Similarly, a marker in BTA 5 (19,823,030 bp), located approximately 109 Kb upstream of an uncharacterized gene (gene ID: 112446651), presented suggestive interaction (*p*-value < 8.9 × 10^−4^) with markers located in intronic or exonic regions of at least 4 different genes of the BTA 21 (*MKRN3*, *MAGEL2*, *SNRPN*, and *GABRG3*).

**FIGURE 7:**
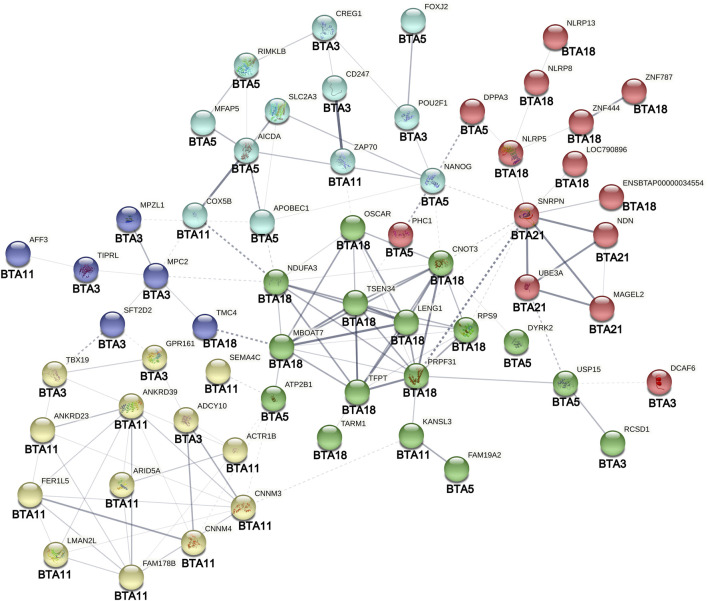
Protein-protein interaction analysis of genes surrounding SNPs involved in significant inter-chromosomal hotspots (*p* < 8.9 × 10^−4^) for age at first calving in Nellore cattle. Different node colors represent genes clustered according to their functional similarity. Edges represent protein-protein associations. The edges thickness represents the interaction confidence degree (the thicker the highest is the confidence). Dotted lines represent interactions between clusters. The original figure was edited for including the autosomes (BTAs) in which the genes are located.

The PPI analysis revealed 128 edges for the 69 genes entered, with roughly 54% of the connections representing moderate to strong evidence according to the database mining (Figure 7). The PPI enrichment analysis was statistically significant (*p*-value < 1 × 10^−6^), indicating that the resultant gene transcripts are at least partially biologically connected and interact more than expected for a random set of proteins of the same size. Although the statistical interactions found in this study were not directly confirmed by the PPI network, many genes flagged by SNPs with significant inter-chromosomal epistasis appeared in the network with connections involving the same set of autosomes. For instance, the *CD247* (BTA3) presented the strongest evidence for a functional link with the *ZAP70* (Figure 7), which is located only ∼1.57 Mb distant from the *AFF3* (BTA11). Another noticeable example is the *TBX19*, that formed edges with two other genes located in the BTA11 (*ANKRD39* and *ANKRD23*). In addition, there is strong evidence that the edge *SNRPN*—*PRPF31* connects functionally two clusters of genes located in the BTA21 and BTA18 (Figure 7). Noticeably, some markers located in the chromosome regions covered by these two clusters presented a suggestive interaction with a marker in the BTA5 (Table 1). Moreover, a cluster of genes in the BTA21 (*GABRG3*, *MAGEL2*, *MKRN3*, *NDN*, *SNRPN*, and *SNURF*) was significantly associated (FDR = 1.07 × 10^−6^) with the Prader-Willi and Angelman syndromes pathway in the PPI analysis.

## 4 Discussion

Age at first calving is a complex trait that reflects the heifer’s reproductive performance in at least three different stages, the time to puberty onset, the interval between puberty onset and the first conception, and gestation length. It is a sex-limited trait that presents low to moderated heritability estimates and has polygenic nature, which imposes several limitations on gene mapping (Grossi et al., 2008; Mota et al., 2017; Schmidt et al., 2018). In this regard, GWAS results reported with different methods are expected to provide complementary insights for clarifying the genetic mechanisms involved in AFC expression. Here we performed an RF-based non-parametric GWAS to rank high-density SNP markers according to their average predictive importance, computed in multiple independent runs. This approach enabled us to identify several SNPs within genomic regions sp anning multiple promising candidate genes, some which of them have not been previously reported in GWAS for economically important traits in Nellore cattle.

Remarkably, many lead SNPs are close to at least 11 candidate genes (*SP3*, *DPPA3*, *NANOG*, *GPR161*, *SEMA4C*, *VAX2*, *MYO18B*, *CNOT3*, *NLRP5*, *ATP6V1B1*, and AXIN1) that coordinate biological functions indispensable for embryonic development (Figure 5). These annotated genes are located in 7 different BTAs (2, 3, 5, 11, 17, 18, and 25), illustrating the tremendous complexity of this process. Failure in the pre-implantation stages causes embryo resorption, which delays the interval between the female exposure and the successful calving. Not surprisingly, most of these genes are required for embryo viability after conception and play critical roles in early and later developmental processes. For instance, the *SP3* gene, a member of the SP1-like transcription factors family (Zhao and Meng, 2005), has ubiquitous expression in early embryos, and its knockout is associated with growth retardation and death at birth in mice (Bouwman et al., 2000). This gene is also required for skeletal ossification in mice (Gollner et al., 2001; Pichel et al., 2003) and for enhancing the ability of embryonic stem cells to differentiate into osteoblasts (Gollner et al., 2001).

Located in the *Bos taurus* autosome 5*,* the *DPPA3* (Developmental pluripotency-associated 3) has been found to present high expression in the oocyte of human primordial follicles (Markholt et al., 2012) and female mice embryonic gonads at 18.5 days after breeding (Small et al., 2005). This is a maternal effect gene that regulates normal development in mice during the embryo preimplantation stage. It has been detected in primordial germ cells, oocytes, preimplantation embryos, and pluripotent cells (Payer et al., 2003). Similarly, *NANOG* and its paralog *NANOGNB* are highly expressed during embryo preimplantation stages in humans, mice, and cows (Dunwell and Holland, 2017). Therefore, it is clear that these pluripotency cell-associated neighboring genes (*DPPA3* and *NANOG*) have a co-expression role in the bovine embryo pre-implantation process (see Figure 7), probably also coordinating cell differentiation after embryo fertilization (Dunwell and Holland, 2017).

The *CNOT3* and *NLRP5*, located in the BTA 18, were found close to the *BovineHD1800018414* (63.43 Mb). The CCR4-NOT transcription complex subunit 3 (*CNOT3*) is a transcription activity regulator, this gene was flagged by SNPs validated for fertility (pregnancy within the first 42 days of mating) in two distinct dairy breeds (Pryce et al., 2010). In mammals, the *CNOT3* may have roles in embryonic viability, since a deficiency in this gene resulted in lethality at early embryonic stages in mice (Morita et al., 2011). Interestingly, interactions between the *NANOS2* gene (Nanos C2HC-Type Zinc Finger 2) and the CCR4-NOT deadenylation complex (including *CNOT3*) play an essential role in male germ cell development in mice (Suzuki et al., 2012). The *NLRP5*, also known as the maternal antigen that the embryo requires (MATER), integrates the subcortical maternal complex, an essential multiprotein complex for embryonic development and uniquely expressed in mammalian oocytes and early embryos (Bebbere et al., 2016). Additionally, other genes of the *NLRP* subfamily stand side by side with the *NLRP5*, the *NLRP8* initializes approximately 28 Kb upstream to the *BovineHD1800018414* marker, whereas the *NLRP13* is flagged at an intronic region by this same SNP. These are oocyte- or germ-cell-specific syntenic genes required for the normal operation of mammalian reproductive systems (Tian et al., 2009).

The *AX1N1* in the BTA 25 is also required for normal embryogenesis, it is known that the complete inactivation of this gene results in early embryonic lethality in mice, caused by different development defects such as forebrain absence and embryonic axis duplications (Zeng et al., 1997; Chia et al., 2009). Furthermore, a cluster of genes (*HBZ*, *HBA*, *HBM*, *HBQ1*, *RFPS9*, and *NARFL*) in this same chromosome may have complementary functions in embryo development regulation through hemoglobin complex-related pathways (Figure 5)*.* Hemoglobin (Hb) is mainly found in erythrocyte cells; however, there is recent evidence of ovarian regulation of Hb synthesis through the ovulatory signal cascade, with high expression of Hb subunits in human granulosa and cumulus cell samples, suggesting a potential role of the hemoglobin complex in the early embryo development (Brown et al., 2015).

Another interesting biological pathway involving the genes *LOC107131465*, *LOC107131469*, *LOC107131476*, and *LOC107131477* (all located in the BTA 18) was associated with pheromone receptor activity (Figure 5). Pheromone activity influences sexual behavior and reproductive hormone secretion in different species. Although the role of pheromone in cattle reproduction is not fully understood, there are shreds of evidence that beef heifers attain puberty faster when exposed to the male presence (Oliveira et al., 2009; Fiol et al., 2010). Fiol and Ungerfeld (2016) reported that exposing anestrous heifers to androgenized steers promoted an increase in basal levels of LH after 10 days of exposure. Therefore, the high frequency of favorable alleles involved in pheromone recognition is particularly interesting in extensive beef production systems, where females are exposed to bulls during the breeding season.

Other candidate genes found in this study are necessary to regulate biological functions related to male fertility maintenance. For instance, the *adenylated cyclase 10* (*ADCY10*) has a critical role in sperm maturation in the epididymis, this gene is located in the BTA 3 and downstream the marker *BovineHD0300000287*. It was noticed that splicing errors in the *ADCY10* were responsible for bovine spermatozoa subfertility (Noda et al., 2013), whereas the orthologous version of *ubiquitin Specific Peptidase 15* (*USP15*) in mice is expressed in the developing acrosomal cap of spermatids in the testes (Crimmins et al., 2009). Besides *ADCY10* and *USP15*, expressed in male germ cells, there are other annotated genes previously associated with bull fertility traits, such as *FUT8* (located in the BTA 10), significantly associated with sire conception rate (Rezende et al., 2018), and *NOB1* and *NFTA5* (located in the BTA 18), found in whole-exome sequencing of bulls divergent for fertility (Whiston, 2017). Furthermore, some genes identified in the BTA 25 may have a deleterious role on male andrological parameters, such as the *Calpain-15* (*CAPN15*), which has a causal variant affecting cryptorchidism susceptibility in rats (Barthold et al., 2016). It is biologically plausible that several genes have pleiotropic effects on both male and female fertility traits, for instance, the *AXIN1* is requested for successful embryo development (Xie et al., 2011) and has been shown to act as a suppressor of testicular germ cell tumors (Xu et al., 2017). Similarly, the *APOBEC1* controls testicular germ cell tumor susceptibility and embryonic viability through transgenerational epigenetic inheritance (Nelson et al., 2012). These findings corroborate the favorable genetic correlations between male and female reproductive traits reported for beef cattle (Terakado et al., 2015).

There is also statistical evidence of the genetic association between reproductive and growth traits in Nellore and other cattle breeds (Santana et al., 2012; Caetano et al., 2013, Pires et al., 2017). Part of our findings reinforces biologically these estimated associations as some genomic regions highlighted here have been previously associated with growth-related traits in Nellore cattle. In the BTA3, the RF analysis pointed to an LD block of several neighboring SNPs associated with AFC, located between positions 0.262 and 1.207 Mb and harboring a total of 30 candidate genes (Supplementary Table S1), including the *POU2F1* and *CREG1*, two transcription factors that integrate metabolic pathways for the regulation of muscle and fat tissues development (Pérez-Montarelo et al., 2014; Hashimoto et al., 2019). This genomic region in the BTA3 encompasses a 1 Mb length window, previously associated with muscling and conformation scores in Nellore cattle (Carreño et al., 2019). Further, the *BovineHD0500014854* marker (51.43 Mb) found in the BTA 5 (Supplementary Table S1) is located at an intronic region of the *FAM19A2* gene, which was previously identified using Bayesian inference within a 1 Mb length window that explained 1.78% of the additive genetic variance for weight gain from birth to weaning in Nellore cattle (Terakado et al., 2017). These results suggest that these regions in BTAs 3 and 5 span QTLs with pleiotropic effects in reproductive, growth, and muscle development traits in Nellore cattle. This hypothesis is strengthened by the fact that the *POU2F1* integrates the interactome associated with the control of embryonic stem cell pluripotency (Ferraris et al., 2011, see also Figure 7) and that the *CREG1* promotes cardiomyogenesis in the mouse embryo, with its genetic ablation resulting in embryonic lethality (Liu et al., 2016).

Among the SNPs with the highest importance scores, four markers were located in the BTA21, between 0.81 and 2.17 Mb (Supplementary Table S1). This region harbors the *SNRPN*, *SNURF*, *MAGEL2*, *MKRN3*, and *NDN* imprinted genes, which have well-known roles in epigenetic regulation of precocious puberty onset, reproductive hormones synthesis, oocytes development, and, pre or post-implantation of embryos in cattle and humans (Suzuki et al., 2009; Piedrahita, 2011; O’Doherty et al., 2012; Abreu et al., 2013; Duittoz et al., 2016). This LD block highlighted in BTA21 also encompasses the *UBE3A* gene, ranked as third in the functional prioritization analysis (Supplementary Table S2). The *UBE3A* is a maternally imprinted gene that encodes the E3 ubiquitin ligase protein and is also responsible for coactivating steroid hormone receptors, including estrogen (*TFF1* and *GREB1*), progesterone (*PGR*), and androgen (*KLK3*) receptor responsive genes (Nawaz et al., 1999; Khan et al., 2006; Catoe and Nawaz, 2011).

Using the single-step GBLUP (ssGBLUP) approach, the same set of genes found in the BTA21 was reported for early pregnancy in Nellore cattle, in a study that used partially the same dataset as in the present work (Irano et al., 2016). These authors noticed that a window comprising the genomic region between 8,725 and 3,028,689 bp in the BTA21 (which flanks the SNPs with the highest importance scores in the present study) was responsible for the largest genetic variance explained (1.31%) for early pregnancy. The *MAGEL2* gene region was also previously associated with the scrotal circumference in Nellore cattle, similarly, the genomic region spanning this gene explained the highest proportion of the additive genetic variance (Utsunomiya et al., 2014). Hence, the empirical evidence provided so far points out that the SNPs identified in our study in the BTA21 (between 0.81 and 2.17 Mb) are in LD with single or multiple QTLs presenting major effects for fertility-related traits in Nellore cattle.

It is also worth noting that other candidate genes annotated near relevant SNPs in our study have been validated in different dairy cattle populations. The *ATPase Ca++ transporting plasma membrane 1* (*ATP2B1*) ends approximately 215 Kb downstream of the marker *BovineHD0500005765* (19.82 Mb). This same gene was located in the vicinities of single nucleotide polymorphisms significantly associated with calving interval in Italian Holstein Cattle (Minozzi et al., 2013). Besides *CNOT3* and *NLRP5*, flagged by the SNP *BovineHD1800018414* in the BTA18 (63.43 Mb), the *RPS9* may also have an important role in the regulation of AFC. This gene is located at an intronic region of a putative QTL for calving traits (calving ease, calf size, stillbirth, birth index, body depth, and stature) segregating in Holstein cattle at approximately 57 Mb (Mao et al., 2016). Additionally, eight sequence variants of the *RPS9* had the strongest associations with fertility traits (*p* < 1 × 10^−10^) in dairy cattle and, at the same time, exhibited lesser expression in the corpus luteum of low fertility cows (Moore et al., 2016).

In summary, an extensive literature search revealed that many annotated genes have well-known functions associated with embryo pre-implantation, embryonic development, male fertility, synthesis of reproductive hormones, and pheromone recognition. Some genomic regions identified in BTA3 and BTA5 in the present study were previously associated with weight gain from birth to weaning and visual scores at weaning in Nellore cattle (Terakado et al., 2017; Carreño et al., 2019); these traits are closely related to heifers body condition before puberty onset. In beef cattle, high body size delays the puberty onset, whereas animals with high weight-height ratios at 11 months of age are expected to have low age at puberty (Pereira et al., 2017). Therefore, genes with important roles in the regulation of growth traits are expected to influence fertility as well. Also, a genomic region strongly associated with fertility-related traits in Nellore cattle, validated with different methods and in different populations (Utsunomiya et al., 2014; Irano et al., 2016) was also highlighted in the present study, which reinforces RF effectiveness for pre-screening candidate QTLs associated with complex traits. Nevertheless, some regions significantly associated with AFC in previous studies were not identified here, for instance, the genomic region surrounding the *PLAG1* in the BTA14 (Mota et al., 2017). This lack of replication between trait-associated markers in the same breed may be due to data particularities such as sample size, the extent of LD, minor allelic frequency, population structure, and also due to potential false discoveries and the different data analysis methods employed.

Most of the standard parametric methods for genome-wide scans focus only on the additive allelic substitution effect, whereas genomic variants involving hidden non-linear patterns within or between *loci* remain overshadowed. Mapping epistatic interactions in high-density SNP data is both statistically and computationally challenging because testing every first order epistatic interaction reduces drastically the statistical power due to multiple testing penalization, while exponentially increasing the computational runtime. These challenges explain the relatively small number of epistatic loci reports for complex traits in livestock species.

Here, the associations between the markers and the response variable were investigated under a non-parametric approach. It has been shown that the tree-based ensemble in the RF can implicitly capture the additive effects and possible non-linear genetic associations between the markers and phenotype, e.g., dominance and epistasis (Garcia-Magarinos et al., 2009; Yao et al., 2013; Alves et al., 2020). Epistatic interactions between markers are adaptatively captured in the RF during the tree recursive splitting process so that SNP pairs that jointly present a large interaction effect will appear more frequently as a parent-child node in the same branch of a tree (Yao et al., 2013). Following this assumption, pairwise interactions were tested between the relevant SNPs pre-selected in the RF genome-wide scan for AFC, treating the marginal and epistatic effects as fixed in linear mixed models. These complementary analyses revealed that many SNPs highlighted in the RF approach present relatively small additive effects in the linear model but are potentially interacting with other markers in different chromosomes. The markers with weak marginal effects would possibly not surpass standard *p*-values threshold criteria adopted in GWAS performed with traditional linear parametric models. This fact shed light on the importance of aggregating the complementary biological knowledge obtained with different methodologies.

As an alternative to reducing the statistical and computational complexities of testing multiple interactions some authors propose to test epistatic effects only between SNPs with the highest −log_10_ (*p*-values) in standard GWAS and the remaining markers (Bolormaa et al., 2015) or between SNPs surpassing a nominal *p*-value threshold for the additive effects, e.g., 0.01 (Ali et al., 2015). Although these approaches are interesting, they present some drawbacks. For instance, it is assumed that all markers involved in significant epistatic interactions are also expected to present some marginal additive effect, which is not always the case. In the RF algorithm, all SNPs are included simultaneously in the analysis, allowing one to identify markers involved in potential interaction networks rather than in isolated pairwise interactions. This can be illustrated by the multiple epistatic interactions found between a marker in BTA 11 at an intronic region of the gene *AFF3* and markers located within the genes *CD247*, *DCAF6*, *TBX19*, and *MPZL1*, all located in the BTA 3.

A common argument in animal breeding theory towards the relative unimportance of the epistatic gene action in complex traits is that the main source of the genetic variance observed in field data is mostly additive (Crow, 2010; Hill, 2010). It is known that additive variance can arise in highly non-linear systems (Hill et al., 2008) and the opposite can also be true, i.e.*,* models parametrized consistently with non-additive gene action could capture most of the genetic variance, even when the genetic architecture is purely additive (Mackay 2013; Huang and Mackay, 2016; Sackton and Hartl, 2016). These results illustrate why generally one cannot infer the prevalent gene action of complex traits based on observational variance components results (Huang and Mackay, 2016).

In this study, there was evidence that epistatic interactions at the level of individual genotypes can be associated with EBVs for AFC in Nellore cattle, which theoretically rely only on additive signals. Interacting loci can generate a substantial genetic additive variance for a wide range of allele frequencies, especially when the MAF of at least one locus is low (Mackay 2013; Huang and Mackay, 2016), this is especially the case in populations under directional genetic selection. Consequently, the epistatic interaction effects are “converted” in standard linear models assuming infinitesimal additive contribution. Hence, ignoring epistatic gene action in the model generally has little consequences if the interest is to estimate heritability, predict breeding values, or infer short-term response to artificial selection (Crow 2010). However, physiological epistasis (i.e., at the level of individual *loci*) is independent of the interacting *loci* allele frequencies and its knowledge may present importance to dissecting the genetic architecture of complex traits and understanding the biological function of candidate genes (Mackay 2013).

In the linear mixed models (M1 and M2) used to investigate the genetic effects of markers identified in the RF genome-wide scan, the random polygenic component accounted for approximately 54% of the total observed variance in the breeding values. This result matches the pedigree-based EBV expected accuracy for the reference population so that the main and interaction effects were captured as extra hidden variation in the residual component. This is important to avoid potential confounding with the additive covariance structure present in the data.

Nonetheless, caution is required to infer the significant interactions observed in this study as causal gene-gene epistatic effects, since imperfect LD between a marker pair and the causal QTL can create the illusion of the presence of non-linearity in purely additive systems, the so-called phantom epistasis (de los Campos et al., 2019). However, the phantom epistasis phenomenon occurs predominantly between physically close *loci* (de los Campos et al., 2019) whereas all relevant interactions reported in this study involve markers in different chromosomes.

One possible explanation for why the detected interacting marker pairs were mostly located in different autosomes lies in how the RF algorithm operates and its limitations toward the presence of highly correlated variables in the dataset. The RF importance scores are computed by measuring the prediction error increase when a particular variable is randomly permuted in the OBB data. During the tree-building process, a highly correlated marker is very unlikely to be the best variable to split on the child nodes whereas epistatic SNP pairs appear more frequently as a parent-child node within the same tree (Yao et al., 2013), this reduces the ability of local interactions signals being captured within the tree ensemble. Furthermore, the presence of high LD for loci located very close may reduce the power for detecting markers with weak to moderate additive or local epistatic effects, since linked loci can serve as surrogates for each other. Strictly speaking, the RF importance score for any causal locus will be diluted through highly correlated markers if they remain unshuffled in the same tree, although this bias is more prominent for importance measures based on the Gini-index (Nicodemus and Malley, 2009). Conversely, the effect generated by the interaction between unlinked loci is more easily broken if one of the interacting markers is shuffled within the tree, therefore, increasing the importance score for the markers involved in intergenic interactions.

It is noteworthy that interactions between unlinked genes are biologically supported if the involved loci encode components of a metabolic pathway or network, signaling pathway, or transcription factor network (Phillips, 2008). Moreover, although unlinked, the interacting marker pairs present some low association as evidenced by the GPD estimates computed with the *r* ^*2*^ metric (Table 1). It is known that epistatic interaction can establish and maintain non-random associations between markers at independent *loci* if selection favors certain allelic combinations (Mueller and James, 1983). These anomalous associations could be viewed as further evidence for the existence of real interaction effects between the reported markers. Once again, caution must be exercised since other evolutionary forces such as genetic drift and non-random mating also tend to increase long-range linkage (gametic-phase) disequilibrium (Goddard and Hayes, 2009; Qanbari 2020).

Still, many genes flagged by interacting markers are biologically plausible to be involved in epistatic hotspots. For instance, the *AFF3* gene is a transcription factor that interacts with different zinc finger proteins for the epigenetic regulation of imprinted genes by binding to both differentially DNA-methylated and enhancer regions of mouse embryonic stem cells in an allelic-specific manner (Luo et al., 2016; Wang et al., 2017). The *DCAF6*, one of the candidate epistatic pairs for *AFF3* is also a cofactor that enhances the transcriptional activity of androgen receptors (Tzung-Chieh et al., 2005; Chen et al., 2017) while both *TBX19* and *AFF3* are related to adrenocortical-related dysfunction in humans (Couture et al., 2012; Lefèvre et al., 2015).

The RF approach has been successfully applied for genome-wide scanning in livestock data. For instance, Mokry et al. (2013) applied the RF algorithm to identify a subset of SNPs that explained approximately 50% of the deregressed breeding values for backfat thickness in Canchin beef cattle. Similarly, Yao et al. (2013) examined the most frequently occurring descendent pairs within the RF tree ensemble to identify SNPs with potential epistatic effects for residual feed intake in dairy cattle. More recently, it has been shown that RF is an efficient methodology for sampling an optimal subset of SNPs for genomic prediction of growth traits in beef cattle (Li et al., 2018). Here, we provided further evidence for the usefulness of RF for dissecting biological mechanisms involved in the regulation of complex traits in beef cattle. Thus, RF is an interesting complementary tool to the traditional parametric methods of GWAS.

## 5 Conclusion

To the best of our knowledge, this was the first attempt of applying a non-parametric approach for scanning potential loci affecting reproductive traits in Nellore cattle using high-density genomic data. The RF-based genome-wide scan and functional analysis highlighted genomic regions spanning candidate genes with key roles in fertility, including embryo pre-implantation and development, embryonic viability, male germinal cells maturation, and pheromone recognition. Complementary analyses revealed that many top-ranked markers in the RF-based GWAS did not present a strong marginal linear effect but are potentially involved in epistatic hotspots between genomic regions in different autosomes. The reported results are expected to enhance the understanding of genetic mechanisms involved in the regulation of AFC in this breed.

## Data Availability

The data analyzed in this study is subject to the following licenses/restrictions: The data that support the findings of this study were obtained under license from the Alliance Nellore dataset (www.gensys.com.br) with availability restrictions applied. Data are however available from the authors upon request and with permission of the third parties involved. Requests to access these datasets should be directed to galvao.albuquerque@unesp.brs.
