# Supplementary figures and images for: A Random Forest-Based Genome-Wide Scan Reveals Fertility-Related Candidate Genes and Potential Inter-Chromosomal Epistatic Regions Associated With Age at First Calving in Nellore Cattle
Source: Front Genet. 2022 May 18;13:834724. doi: 10.3389/fgene.2022.834724 (PMC9178659; doi:10.3389/fgene.2022.834724)

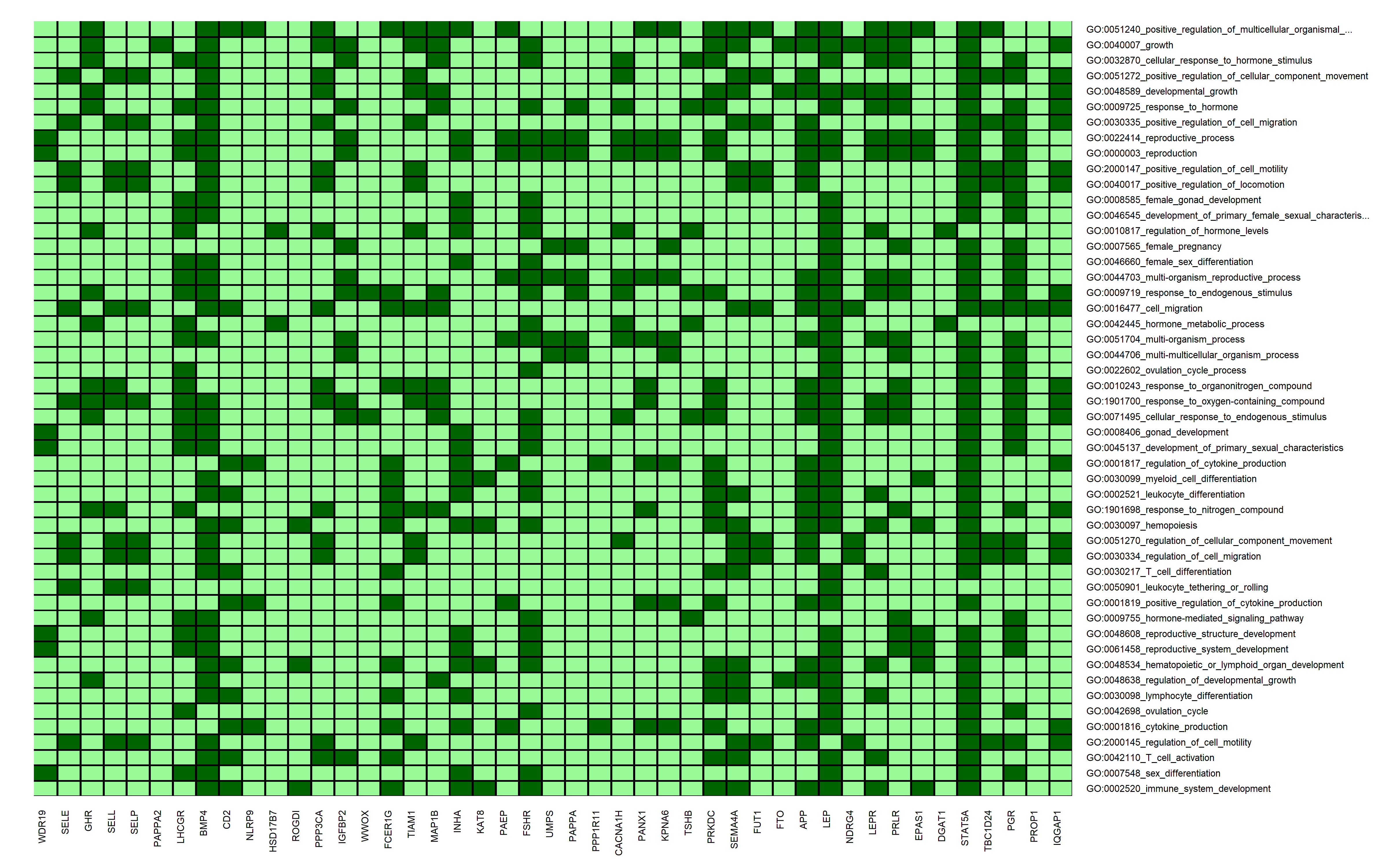

Supplement: Supplementary file 1 [file Image1.TIFF]
