# Supplementary material for: A Random Forest-Based Genome-Wide Scan Reveals Fertility-Related Candidate Genes and Potential Inter-Chromosomal Epistatic Regions Associated With Age at First Calving in Nellore Cattle
Source: Front Genet. 2022 May 18;13:834724. doi: 10.3389/fgene.2022.834724 (PMC9178659; doi:10.3389/fgene.2022.834724)
Supplement: Supplementary file 2 [file Table1.DOCX]

**SUPPLEMENTARY TABLE S1.** Relevant SNPs id­­entified in the Random Forest analysis for age at first calving (AFC) in Nellore cattle.

| **SNP Name** | **BTA** | **Position (Mb)** | **Candidate genes within ± 250 Kbp interval** | $\boldsymbol{f}_{\boldsymbol{SNPj}}$ |
| --- | --- | --- | --- | --- |
| BovineHD0200004124 | 2 | 14.57 | *PDE1A, LOC101905179, PPP1R1C, LOC101907472, SSFA2* | 3.17 |
| BovineHD0200006533 | 2 | 23.02 | *SP3, TRNAA-UGC, LOC105180379, LOC107132247* | 3.60 |
| BovineHD0300000049 | 3 | 0.26 | *LOC787691, LOC787668, LOC112445856, LOC101907782, LOC100336903,* *LOC112445857, LOC100335782, LOC107131930, LOC790004, TBX19, SFT2D2,LOC104971401,TIPRL, GPR161* | 3.31 |
| BovineHD0300000122 | 3 | 0.57 | *DCAF6, LOC104971402, LOC101904335, MPC2, LOC107131946, ADCY10* | 4.23 |
| BovineHD0300000287 | 3 | 1.13 | *MPZL1,TRNAP-AGG,TRNAP-CGG, RCSD1, LOC112445920, LOC104971407 CREG1, CD247, POU2F1, LOC112445922, LOC112445921* | 6.84 |
| BovineHD0500005765 | 5 | 19.82 | *ATP2B1, LOC112446883, LOC112446651* | 3.03 |
| BovineHD0500013341 | 5 | 46.22 | *LOC112446698, DYRK2, LOC112446699, LOC112446700* | 5.26 |
| BovineHD0500014854 | 5 | 51.43 | *USP15, FAM19A2* | 3.73 |
| BovineHD0500029155 | 5 | 101.28 | *A2ML1 , MIR2284R, RIMKLB, MFAP5, TRNAE-UUC, AICDA, APOBEC1, GDF3M, LOC100295782, DPPA3, LOC512775, NANOG, SLC2A3, PHC1, FOXJ2* | 4.13 |
| BovineHD1000022233 | 10 | 77.52 | *LOC112448644, FUT8, TRNAC-GCA* | 7.02 |
| BovineHD1100000982 | 11 | 2.81 | *ARID5A, KANSL3, LOC112448954, FER1L5, LMAN2L, LOC112448735, CNNM4 CNNM3, ANKRD23, LOC112448736, ANKRD39, LOC112448737, SEMA4C, FAM178B, COX5B, ACTR1B, C11H2orf92, ZAP70* | 4.59 |
| BovineHD1100001777 | 11 | 4.97 | *AFF3* | 3.24 |
| BovineHD1100004325 | 11 | 13.23 | *DYSF, LOC112448752, LOC101903215, ZNF638, TRNAC-GCA, PAIP2B, NAGK, LOC112448753, TEX261, ANKRD53, ATP6V1B1, VAX2* | 4.81 |
| BovineHD1100005572 | 11 | 18.13 | *LOC100849080, LOC100140559, LOC112448869, LOC112448951* | 8.05 |
| BovineHD1700019684 | 17 | 65.59 | *LOC112442007, GRK3, MYO18B, LOC112442055, TRNAS-GGA* | 3.22 |
| BovineHD1800018414 | 18 | 63.43 | *LOC107131323, LOC112442224, RPS9, TSEN34, MBOAT7, TMC4, LENG1, CNOT3, LOC101905303, PRPF31, TFPT, NDUFA3, OSCAR, TARM1 LOC107131473, LOC107131475, LOC107131476, LOC107131467, LOC107131477, LOC107131468, LOC107131469, NLRP13, LOC107131465, NLRP8, NLRP5, ZNF787, ZNF444, LOC101903510, LOC112442416, LOC790201, LOC101903702, LOC100140659, LOC100140412, ZSCAN5B, LOC790324, LOC104974965, LOC790896, LOC768229, LOC112442240, LOC112442417, LOC104968608, LOC100848202, LOC112442391,LOC782638 LOC530319, LOC783562, LOC104968500* | 4.83 |
| **­Table S1.** Relevant SNPs id­­entified in the Random Forest analysis for age at first calving (AFC) in Nellore cattle. (Continued) | | | | |
| BovineHD2100000137 | 21 | 0.81 | *LOC112443246, LOC101908683, LOC112443373, LOC112443363, LOC112443211, LOC112443207, LOC112443209, LOC112443210* | 8.54 |
| BovineHD2100000071 | 21 | 1.31 | *MKRN3, NDN, MAGEL2* | 10.37 |
| BovineHD2100000038 | 21 | 1.59 | *-* | 7.59 |
| BovineHD2100000275 | 21 | 2.17 | *LOC112443247, SNRPN, SNURF, LOC100849023, LOC112443214, LOC112443336, LOC112443258, LOC112443280, LOC112443259, LOC112443260, LOC112443276, LOC112443261, LOC112443262, LOC112443263, LOC112443264, LOC112443265, LOC112443266 LOC112443268, LOC112443284, LOC112443278, LOC112443269, LOC112443270, LOC112443271, LOC112443272, LOC112443273, LOC100848941, LOC112443274, LOC112443275, LOC112443277, LOC112443283, LOC112443279, LOC112443281, LOC112443282, LOC112443257,LOC101907203, LOC112443253, LOC112443251, LOC112443252, LOC112443254, LOC112443255, LOC112443338, UBE3A, LOC112443215* | 9.31 |
| BovineHD2100000879 | 21 | 4.93 | *GABRG3, LOC112443369* | 9.17 |
| BovineHD2400018598 | 24 | 55.61 | *LOC112444192, LOC112444186* | 4.04 |
| BovineHD2500000080 | 25 | 0.42 | *NPRL3, HBZ, LOC100137913, HBM, HBA, HBA1, HBQ1, LOC100294963, LUC7L FAM234A, RGS11, ARHGDIG, PDIA2, AXIN1, LOC112444351, MRPL28, LOC112444352, TMEM8A, NME4, DECR2, RAB11FIP3, LOC104975822, CAPN15, PRR35, NHLRC4, PIGQ, RAB40C, WFIKKN1, METTL26, TRNAG-CCC, MCRIP2, WDR90, RHOT2, RHBDL1, LOC516108, STUB1, JMJD8, WDR24, FBXL16, METRN, FAM173A, CCDC78, HAGHL, NARFL, MSLN, LOC531296, RPUSD1* | 3.53 |

BTA, Bos Taurus Autosome

$f_{SNPj}$, SNP relative importance score
