# Supplementary material for: A Random Forest-Based Genome-Wide Scan Reveals Fertility-Related Candidate Genes and Potential Inter-Chromosomal Epistatic Regions Associated With Age at First Calving in Nellore Cattle
Source: Front Genet. 2022 May 18;13:834724. doi: 10.3389/fgene.2022.834724 (PMC9178659; doi:10.3389/fgene.2022.834724)
Supplement: Supplementary file 3 [file Table2.DOCX]

**SUPPLEMENTARY TABLE S2.** Top 30 candidate genes for age at first calving in Nellore cattle ranked according to the functional prioritization analysis.

| Rank | Gene symbol | Gene ID | BTA | Average Score | Overall p-value |
| --- | --- | --- | --- | --- | --- |
| 1 | NDN | 4692 | 21 | 0.79 | 5.78E-04 |
| 2 | AXIN1 | 8312 | 25 | 0.63 | 0.00172 |
| 3 | UBE3A | 7337 | 21 | 0.57 | 0.00465 |
| 4 | MPZL1 | 9019 | 3 | 0.82 | 0.00471 |
| 5 | SP3 | 6670 | 2 | 0.65 | 0.00494 |
| 6 | TBX19 | 9095 | 3 | 0.66 | 0.01352 |
| 7 | ZAP70 | 7535 | 11 | 0.58 | 0.01585 |
| 8 | USP15 | 9958 | 5 | 0.61 | 0.01882 |
| 9 | SEMA4C | 54910 | 11 | 0.53 | 0.01983 |
| 10 | CD247 | 919 | 3 | 0.56 | 0.02032 |
| 11 | SNRPN | 6638 | 21 | 0.58 | 0.02047 |
| 12 | ATP2B1 | 490 | 5 | 0.62 | 0.02065 |
| 13 | GABRG3 | 2567 | 21 | 0.46 | 0.02089 |
| 14 | TFPT | 29844 | 18 | 0.46 | 0.02328 |
| 15 | MAGEL2 | 54551 | 21 | 0.56 | 0.02469 |
| 16 | STUB1 | 10273 | 25 | 0.53 | 0.02470 |
| 17 | APOBEC1 | 339 | 5 | 0.63 | 0.02914 |
| 18 | NPRL3 | 8131 | 25 | 0.45 | 0.03196 |
| 19 | AFF3 | 3899 | 11 | 0.63 | 0.03451 |
| 20 | TEX261 | 113419 | 11 | 0.61 | 0.03457 |
| 21 | RAB40C | 57799 | 25 | 0.39 | 0.03763 |
| 22 | POU2F1 | 5451 | 3 | 0.59 | 0.04292 |
| 23 | DYRK2 | 8445 | 5 | 0.66 | 0.04354 |
| 24 | GPR161 | 23432 | 3 | 0.58 | 0.04374 |
| 25 | MPC2 | 25874 | 3 | 0.59 | 0.04537 |
| 26 | LMAN2L | 81562 | 10 | 0.28 | 0.04543 |
| 27 | FUT8 | 2530 | 5 | 0.48 | 0.04565 |
| 28 | NLRP5 | 126206 | 18 | 0.65 | 0.04600 |
| 29 | ACTR1B | 10120 | 11 | 0.59 | 0.04767 |
| 30 | PRPF31 | 26121 | 18 | 0.47 | 0.05076 |
